# *Entamoeba histolytica*: protein arginine transferase 1a methylates arginine residues and potentially modify the H4 histone

**DOI:** 10.1186/s13071-015-0820-7

**Published:** 2015-04-10

**Authors:** Jessica Borbolla-Vázquez, Esther Orozco, Abigail Betanzos, Mario A Rodríguez

**Affiliations:** Departamento de Infectómica y Patogénesis Molecular, Centro de Investigación y de Estudios Avanzados del IPN, A.P. 14-740, México, D.F. 07000 Mexico

**Keywords:** *Entamoeba histolytica*, Arginine methylation, Protein arginine methyltransferase, Histone modifications, Epigenetics

## Abstract

**Background:**

In eukaryotes, histone arginine methylation associates with both active and repressed chromatin states depending on the residues involved and the status of methylation. Even when the amino-terminus of *Entamoeba histolytica* histones diverge from metazoan sequences, these regions contain arginine residues that are potential targets for methylation. However, histone arginine methylation as well as the activity of arginine methyltransferases (PRMTs) has not been studied in this parasite. The aim of this work was to examine the dimethylation of arginine 3 of H4 histone (H4R3me2) and to identify the parasite PRMT that could be responsible for this modification (EhPRMT1).

**Methods:**

To examine the presence of H4R3me2 in *E histolytica*, we performed Western blot and immunofluorescence assays on trophozoites using an antibody against this epigenetic mark. To recognize the PRMT1 enzyme of this parasite that possibly perform that modification, we first performed a phylogenetic analysis of *E. histolytica* and human PRMTs. RT-PCR assays were carried out to analyze the expression of the putative PRMT1 genes. One of these genes was cloned and expressed in *Escherichia coli*. The recombinant protein was tested by its recognition by an antibody against human PRMT1 and in its ability to form homodimers and to methylate commercial histones.

**Results:**

The arginine 3 of human H4, which is subjected to post translational methylation, was aligned with the arginine 8 of *E. histolytica* H4, suggesting that this residue could be methylated. The recognition of an 18 kDa nuclear protein of *E. histolytica* by an antibody against H4R3me2 confirmed this assumption. We found that this parasite expresses three phylogenetic and structural proteins related to PRMT1. Antibodies against the human PRMT1 detected *E. histolytica* proteins in cytoplasm and nuclei and recognized a recombinant PRMT1 of this parasite. The recombinant protein was able to form homodimers and homotetramers and displayed methyltransferase activity on arginine 3 of chicken H4.

**Conclusion:**

All these results suggest that *E. histolytica* contains as a minimum one structural and functional protein ortholog to PRMT1, enzyme that potentially dimethylates H4R8. This modification may play an important role in the gene expression regulation of this microorganism.

## Background

Arginine methylation is a posttranslational modification occurring in several cytoplasmic and nuclear proteins that modulates chromatin modification (leading to transcription activation or repression), RNA processing, DNA repair, organelle biogenesis, and signaling, among others [1,2]. Arginine methylation involves the transfer of methyl groups from S-adenosyl-methionine (AdoMet) to arginine residues of proteins, and it is catalyzed by enzymes known as protein arginine methyltransferases (PRMTs). PRMTs are classified in four types (I to IV) according to the modification that they generate [2]. Type I PRMTs catalyze the addition of one and two methyl groups to the terminus ω-nitrogen of arginine, producing monomethyl arginine (MMA) and asymmetric dimethylarginine (ADMA). Type II PRMTs also synthesize MMA in the terminus ω-nitrogen, but add a second methyl group to the adjacent terminus ω-nitrogen to yield symmetric dimethylarginine (SDMA). Type III PRMTs catalyze only the production of MMA, and type IV PRMTs catalyze MMA, but on the δ-nitrogen of arginine. Nine PRMTs (PRMT1–9) have been identified in humans; PRMT1, PRMT2, PRMT3, PRMT4, PRMT6 and PRMT8 belong to type I, whereas PRMT5 and PRMT9 showed type II activity [2,3]. PRMT7 has type III activity [4] and type IV PRMTs have been described only in fungi [5,6]. However, the current understanding of arginine methylation in parasitic protozoa is limited (reviewed by Calixto-Galvez, *et al.* [7]).

PRMT1 is the most conserved PRMT, with sequence similarity higher than 90% among vertebrates and higher than 70% between humans and budding yeast [4]. This protein has a broad substrate spectrum, and plays a role in various cellular processes, including transcription activation by the asymmetric dimethylation of arginine 3 of histone H4 (H4R3me2) [2,8]. PRMT1 is also a co-activator of some nuclear receptors, as well as various transcription factors [2,8].

*Entamoeba histolytica* is the protozoan parasite that infects up to 50 million people worldwide each year, causing 40,000 to 100,000 deaths annually [9]. Virulence degree displayed by trophozoites and the life cycle of this parasite must be modulated by changes in gene expression. However, mechanisms involved in gene expression are poorly comprehended in *E. histolytica*. Epigenetics, which refers to chemical changes as DNA methylation and covalent modifications of histones (acetylation, methylation, phosphorylation, ubiquitination, etc.) that result in alterations in chromatin structure and accessibility to the transcription machinery, has been scantily studied in this parasite. The presence of methylated cytosines in *E. histolytica* genome has been established and this modification is catalyzed by a DNA methylase belonging to the Dnmt2 protein family [10]. *E. histolytica* chromatin is organized into nucleosome-like structures [11] and histone encoding genes have been identified and characterized (review by Gomez *et al*. [12]). Interestingly, *E. histolytica* histones belong to the most divergent histone proteins described up to now [12]. For instance, its H4 histone (EhH4) has 71% sequence identity with the consensus sequence, with an insertion of 16 residues in its N-terminus containing several lysine and arginine residues susceptible to be acetylated and/or methylated [13]. Interestingly, acetylation status of lysine residues of histone H4 differs among strains of *E. histolytica* with different virulence degree, suggesting a relationship between H4 acetylation and virulence [14]. The *E. histolytica* genome contains two histone acetylases from GNAT and MYST families and one histone deacetylase of class I [15]. On the other hand, the histone methylation in the lysine 4 of *E. histolytica* histone H3 (H3K4) has been demonstrated by immunodetection [16] and transcriptional silencing has been related to unmethylated H3K4 [17]. It has been defined that this parasite has four putative lysine methyltransferases and five putative PRMTs (EhPRMTs) [7,18]. Fisk and Read [18] reported that one EhPRMT showed homology to PRMT5 and the remaining four displayed limited homology to PRMT1. However, their expression, location and activities have not yet been demonstrated.

In this work we show that an antibody against H4R3me2 recognized a nuclear protein of *E. histolytica*, indicating that this microorganism has an equivalent epigenetic mark. We also demonstrated that *E. histolytica* trophozoites express three PRMTs with structural homology to human PRMT1 (HsPRMT1). Antibodies against HsPRMT1 detected *E. histolytica* proteins in cytoplasm and nuclei and recognized a recombinant EhPRMT1 that is able to form homo-oligomers and displayed methyltransferase activity on the nuclear fraction of trophozoites and on chicken H4. All these results together demonstrate that *E. histolytica* contains at least one structural and functional protein ortholog to PRMT1.

## Methods

### Cell cultures

Trophozoites of *E. histolytica* clone A (strain HM1:IMSS) [19] were axenically cultured at 37°C in TYI-S-33 medium and harvested from confluent cultures as described [20].

Human cervical carcinoma (HeLa) cells were cultured in DMEM (Life Technologies) supplemented with 10% fetal bovine serum. Cultures were incubated at 37°C in a humid atmosphere of 5% CO_2_.

### Isolation of total, cytoplasmic and nuclear extracts from cell cultures

To obtain total proteins, cells (trophozoites or HeLa) were harvested, washed and suspended in cold sterile PBS in the presence of a mixture of a protease inhibitor cocktail (Sigma P2714) plus PMSF 100 mM, PHMB 100 mM and E-64 10 μg ml^−1^. Subsequently, to lyse the cells, samples were submitted twice to freezing in liquid nitrogen and vigorously stirred in vortex until they were completely thawed.

To obtain cytoplasmic and nuclear fractions, cells were resuspended in 2 ml of extraction buffer (10 mM HEPES pH 7.2, 24 mM KCl, 10 mM MgCl_2_, 1 mM PMSF, 2 mM DTT and 0.03% NP-40 (Sigma)) in the presence of a protease inhibitor cocktail (Sigma P2714). After gentle shaking for 30 min, samples were added to a 0.8 M sucrose solution and centrifuged at 6000 × *g* for 10 min at 4°C. The supernatant corresponded to the cytoplasmic fraction, whereas the pellet, corresponding to the nuclear fraction, was resuspended in 2 ml of extraction buffer. Then, to obtain basic proteins, nuclear fraction was incubated 1 h at 4°C with 400 μl of 0.2 N sulfuric acid, and then centrifuged at 13000 × g for 10 min at 4°C. Supernatant was precipitated overnight at 4°C with 25% TCA and 2% acetone. After that, samples were centrifuged at 13000 × g for 10 min at 4°C and pellet of basic proteins was resuspended in 200 μl of distilled water. Protein concentration was determined by the Bradford method [21] and proteins were analyzed by 15% SDS-PAGE.

### Western blot assays

Proteins separated by SDS-PAGE were transferred to nitrocellulose membranes [22] and probed with the primary antibody: anti-H4R3me2 (Abcam) (dilution 1:500); anti-GST (Amersham Biosciences) (dilution 1:5000); or anti-PRMT1 (Cell Signaling) (dilution 1:300). Then, membranes were incubated with the proper peroxidase-coupled secondary antibody (Invtrogen). Finally, reactions were developed by chemiluminescence (ECL Plus, Amersham Biosciences). To demonstrate the enrichment of nuclear and cytoplasmic fractions, these proteins were probed with antibodies against the unmethylated H4 (Santa Cruz Biotechnology) and against the EhCPADH complex [23].

### Immunofluorescence and confocal microscopy

Cells grown on coverslides were fixed and permeabilized with 100% methanol for 5 min and non-specific binding sites were blocked with 1% albumin for 1 h at 37°C. Then, cells were incubated overnight at 4°C with the α-H4R3me2 (1:50) or α-PRMT1 (1:20) antibodies. After several washes, samples were incubated with an anti-rabbit IgG secondary antibody conjugated to FITC (Zymed) (1:100). For co-immunolocalization, α-H4R3me2 and α-PRMT1 antibodies were covalently labeled with Pacific blue 410 and Alexa fluor 647, respectively, using the APEX Antibody Labeling Kit (Life Technologies) according to the manufacturer’s recommendations. Then, cells were incubated overnight at 4°C with these antibodies.

Nuclei were stained with 4′,6-Diamidino-2-Phenylindole (DAPI) and samples were observed through a confocal microscope (Carl Zeiss LSM 700) using the ZEN 2009 software. Observations were performed in approximately 20 optical sections from the top to the bottom of each sample.

### In silico *characterization of E. histolytica PRMTs*

Predicted proteins from the EHI_105780, EHI_152460, EHI_202470, and EHI_159180 gene sequences of *E. histolytica* (http://amoebadb.org) were characterized *in silico* using the software deposited in the Expasy Bioinformatics Resource Portal (http://expasy.org), the NCBI Home Page (http://www.ncbi.nlm.nih.gov), and Biology Workbench software package (http://workbench.sdsc.edu/). Phylogenetic analysis was performed using the Unweighted Pair Group Method with Arithmetic Mean (UPGMA) implemented in the MEGA 5.05 software package [24]. Bootstrapping was performed for 1000 replicates.

### RT-PCR and PCR

Genomic DNA and total RNA were isolated from *E. histolytica* trophozoites using the Wizard Genomic DNA Purification kit (Promega) and the Trizol reagent (Invitrogen), respectively, according to the manufacturer’s recommendations. cDNA was synthesized using an oligo-dT primer and the superscript II reverse transcriptase (Invitrogen).

PCR amplifications were carried out using 200 ng of DNA or cDNA as template and specific primers corresponding to the EhPRMT encoding genes (Table 1). Reactions were carried out in a 50 μl reaction volume containing 0.5 μM of each primer, 2 mM of MgCl_2_, 200 μM of dNTPs, 1X Taq buffer, and 1 U of *Taq* DNA polymerase (Invitrogen). Cycling conditions included an initial denaturation at 94°C for 5 min followed by 28 cycles, with a fast denaturation at 94°C for 1 min, an annealing step for 1 min at 50-55°C, according with the calculated Tm for each fragment, and an extension step at 72°C for 3 min, with a final incubation for 7 min at 72°C. Amplification reaction was followed by electrophoresis on 1% agarose gels. The accuracy of amplified sequences was confirmed by sequencing. As a negative control for PCR experiments we used the same reaction mixture, but without DNA template, whereas for RT-PCR assays we utilized as a negative control the reaction mixture using RNA as template (omitting the retro-transcription step).

**Table 1:** **Primers used to amplify the** ***Ehprmt*** **genes**

**Gene**	**Primer sequences**
**EHI_105780 (** ***Ehprmt1a*** )*	S: 5′-ccccc*ggatcc*atgtcaatggaacaactc-3′
	AS: 5′-ccccc*ctcgag*ttaatttaaatgatattctattttgt-3′
**EHI_152460 (** ***Ehprmt1b*** **)**	S: 5′-atggaagaatgcaaaccaac-3′
	AS: 5′-tcaattcatatgataaacttggtcat 3′
**EHI_202470 (** ***Ehprmt1c*** **)**	S: 5′-atggaatcaaaacaatttg-3′
	AS: 5′-ttattgcatttgatatttcatt-3′
**EHI_159780 (** ***Ehprmt-a*** **)**	S: 5′-atgaaagaaatgaagagaagttt-3′
	AS: 5′-ttaaaattcatattgatattttcctg-3′

S: sense primer. AS: anti-sense primer.

*Sense and anti-sense primers of *Ehprmt1a* contains at their 5′-end the recognition sites for BamH1 and Xho1 (italic), respectively.

### Cloning and expression of the *Ehprmt1a* gene

The full-length *Ehprmt1a* gene (EHI_105780) was PCR-amplified using genomic DNA as a template, and for its directional cloning we used as a sense primer an oligonucleotide containing the BamH1 site before the start codon, and as an anti-sense primer an oligonucleotide that included the Xho1 site before the complementary sequence to the stop codon (Table 1). To prevent the presence of mutations in the amplified product, PCR reaction was performed with the high fidelity enzyme KAPA HiFi DNA polymerase (KAPABIOSYSTEMS). Then, *Ehprmt1a* gene was cloned into the pGEX-6P-1 vector (Amersham Biosciences) using the BamH1 and Xho1 sites.

For *Ehprmt1a* expression, *Escherichia coli* (strain BL21 (DE3)) competent cells were transformed with the pGEX-6P-1 empty vector, used as a negative control, or with the construction containing the *Ehprmt1a* gene. The recombinant proteins (GST and GST/EhPRMT1a) were induced with 0.5 mM IPTG for 3 h at 30°C. Recombinant proteins were purified from bacteria lysates by affinity chromatography using immobilized glutathione (Amersham Biosciences) following the manufacturer’s recommendations. Cleavage of the GST tag was achieved using PreScision protease (Amersham Biosciences, Sweden). Induction of the recombinant protein and its purification was examined by SDS-PAGE.

### Glutaraldehyde cross-linking assay

To determine whether EhPRMT1a forms homo-oligomers, recombinant protein, without the GST tag, was incubated for 10 min at 25°C in 50 mM sodium phosphate (pH 7.4) in a total volume of 30 μl. Then, glutaraldehyde was added to a final concentration of 0.025%, and reactions were allowed to proceed for an additional 10 min at 25°C. Reactions were stopped by the addition of 5 × SDS-PAGE sample buffer and resolved on SDS-PAGE and silver staining.

### PRMT activity assays

PRMT activity assays were analyzed by the incorporation of [^3^H] from [^3^H] AdoMet to histones (HMT assays) as described [25]. The EhPRMT1a recombinant protein (1 μg) was incubated with 10 μg of the nuclear basic fraction of *E. histolytica* or with 0 to 4 μg of histones from chicken erythrocytes (Millipore) and 1 μCi of [^3^H] AdoMet (PerkinElmer Life Sciences) in HMT buffer (50 mM Tris–HCl pH8.0, 5% glycerol, 0.1 mM EDTA, 50 mM KCl, 1 mM DTT, 10 mM sodium butyrate) and in the presence of a protease inhibitor cocktail (Sigma P2714), in a reaction volume of 10 μl. After incubation of 45 min at 30°C, reactions were spotted onto P81 filter circles (Whatman) and allowed to air dry. Then, filters were washed three times with trichloroacetic acid for 15 min, rinsed in ethanol and allowed to air dry. Finally, radioactivity in filters was counted in 4 ml of scintillation fluid (Ready Safe, Beckman Coulter) in a scintillation counter (Beckman LS 6500). These assays were performed three times by duplicate. On the other hand, to determine whether EhPRMT1a methylates the H4R3 residue, we performed HMT assays using reactions containing a mixture of commercial histones (4 μg), non-radioactive AdoMet and 1 μg of the recombinant EhPRMT1a. As a control we performed the reaction in the presence of 1 μg of lysozyme. Finally, reactions were analyzed by Western blot using the α-H4R3me2 antibody.

### Ethical approval

This study was performed in accordance with the guidelines of the Institutional Biosafety Committee, with a previous approval protocol (2014-4-CB). The use of radioactivity was approved by the Institutional Committee on Radiological Protection. Our institution fulfils all the technical specifications for the radioactivity management certified by the Comisión Nacional de Seguridad Nuclear y Salvaguardas de la Secretaria de Energía (licence No. A00.200/0683/2010).

## Results

### *E. histolytica* has an epigenetic mark equivalent to H4R3me2

**Figure 1:**
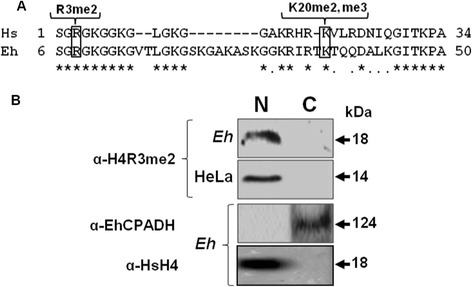
**Identification of an epigenetic mark equivalent to H4R3me2 in** ***E. histolytica*** **. (A)** Alignment of the H4 N-termini of *Homo sapiens* (Hs) and *E. histolytica* (Eh). (*) Identical residues. (**.**) Similar residues. Rectangles indicate arginine and lysine residues susceptible to methylation. **(B)** Western blot using α-H4R3me2 commercial antibody. (N) Nuclear basic proteins and (C) cytoplasmic extracts derived from *E. histolytica* trophozoites and HeLa human cells. As control for cellular fractions, membranes were incubated with α-EhCPADH, antibody against a cytoplasmic protein of *E. histolytica* and with α-HsH4, antibody against unmethylated human H4, used as a nuclear marker.

Alignment of EhH4 with the human H4 showed that residues subjected to post translational methylation, such as the arginine in position 3 and lysine in position 20 (corresponding to positions 8 and 36 in EhH4, respectively) are conserved (Figure 1A). These data suggest that methylation of these residues could also occur in EhH4, and modifications might have a role in the epigenetic regulation of this pathogen.

To investigate whether methylation takes place in EhH4R8, we performed Western blot assays on nuclear basic proteins and cytoplasmic extracts of trophozoites using a commercial antibody against H4R3me2. The antibody recognized an 18 kDa band in nuclear proteins of *E. histolytica* and a 14 kDa band in HeLa cells, used as a positive control (Figure 1B). Antibody did not detect any band in cytoplasmic extracts of *E. histolytica*. An antibody against unmethylated H4, used as a nuclear marker, recognized an 18 kDa band only in nuclear extracts, whereas a 124 kDa band was recognized only in cytoplasmic fraction when we used the α-EhCPADH antibody as a cytoplasmic marker (Figure 1B).

**Figure 2:**
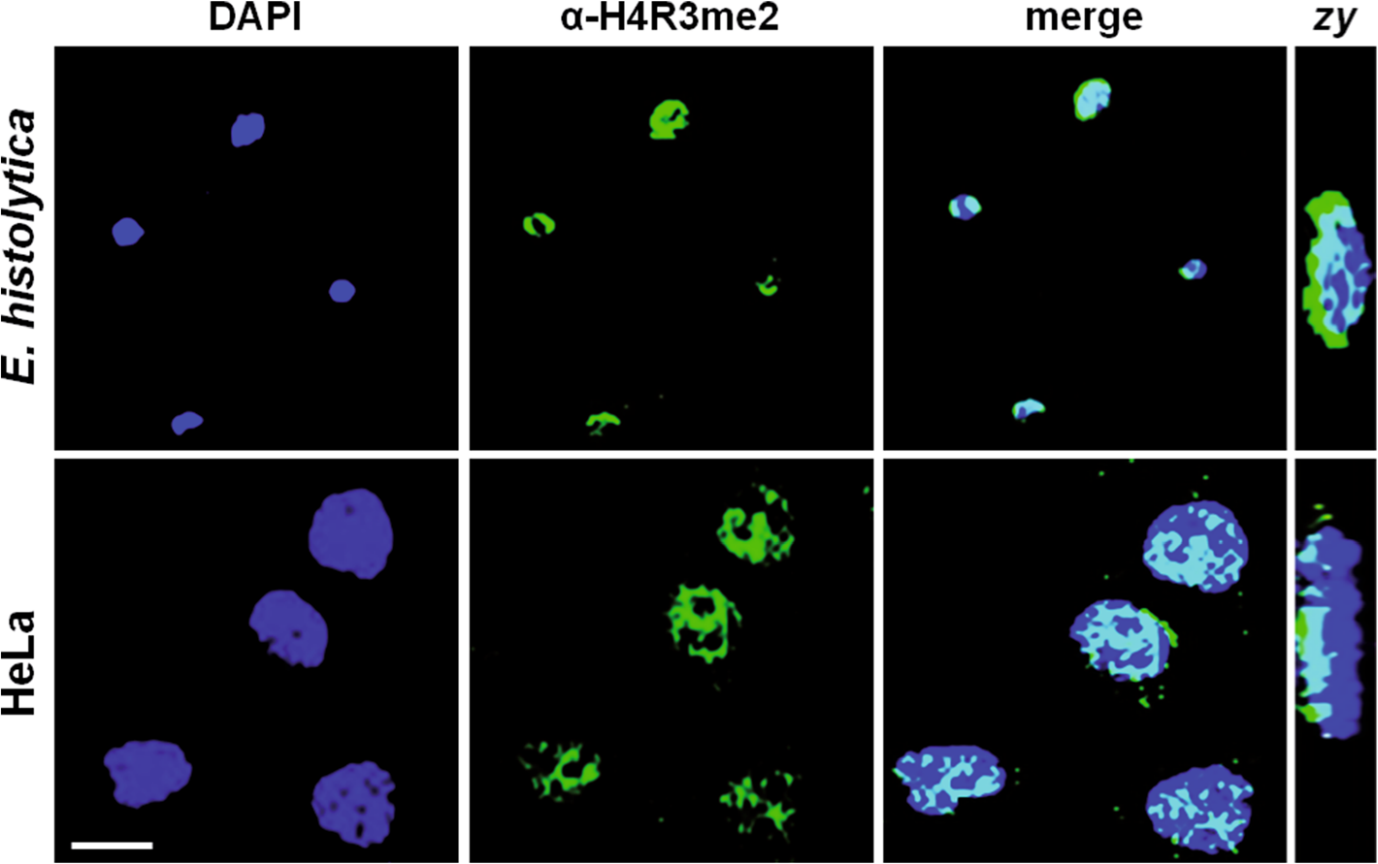
**Cellular localization of H4R8 2me epigenetic mark in** ***E. histolytica trophozoites.*** *E. histolytica* and HeLa cells (positive control) were processed for immunofluorescence assays using the α-H4R3me2 commercial antibody and a FITC-labeled secondary antibody (green). Nuclei were stained with DAPI (blue). *Zy* planes, lateral images of the cells. Bar scale = 10 μm.

Confocal immunolocalization experiments using the α-H4R3me2 antibody also revealed the presence of this epigenetic mark in *E. histolytica* trophozoites. Images showed the antibody recognition in DAPI stained nuclei of trophozoites and HeLa cells, while no mark was detected in the cytoplasm (Figure 2). Z*y* planes confirmed the presence of the epigenetic mark in nuclei and suggested that the EhH4R8 residue (equivalent to the consensus H4R3 in other organisms) probably is methylated. This posttranslational modification may be involved in the epigenetic regulation of *E. histolytica*.

### *E. histolytica* contains and expresses Ehprmt1-like genes

In other eukaryotes, methylation of H4R3 is catalyzed by PRMT1 [2,8]. It has been described that *E. histolytica* contains five putative PRMTs, one with similarity to PRMT5 (EHI_158560) and four PRMTs with similarity to PRMT1 (EHI_159180, EHI_105780, EHI_152460, and EHI_202470) [18]. However, we cannot attribute the methylation of H4R8 showed in Figures 1 and 2 to a particular EhPRMT yet.

**Figure 3:**
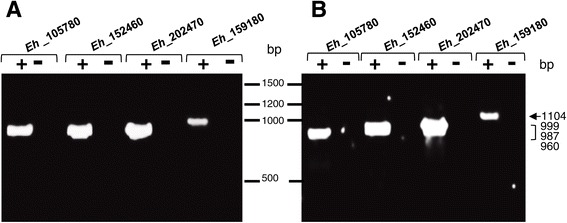
**Genes and transcripts of EhPRMTs. (A)** Genomic DNA or **(B)** RNA obtained from *E. histolytica* trophozoites were subjected to PCR and RT-PCR assays respectively. Then, amplicons were separated in 1% agarose gels. Lanes (+): reactions using genomic DNA or cDNA as template. Lanes (−): reactions without genomic DNA **(A)** or using RNA as template (omitting the cDNA synthesis) **(B)**. Numbers at center: DNA ladder. Numbers at right: molecular sizes of amplified products.

To experimentally probe the existence of the four putative *prmt1* genes reported in *E. histolytica* genome, we carried out PCR assays using specific primers for each gene. These experiments showed the amplification of DNA fragments with the expected size for each putative *Ehprmt1-*like gene: 960 (EHI_159180), 987 (EHI_105780), 999 (EHI_152460), and 1104 (EHI_202470) bp (Figure 3A). RT-PCR assays using the same primers and cDNA as template showed that the four *Ehprmt1-like* genes are expressed in trophozoites (Figure 3B). These results indicated that predicted *Ehprmt1-*like genes indeed exist and are expressed in *E. histolytica* trophozoites. It is predictable that they should be involved in methylation of histones and other proteins that may participate in several cellular pathways, in a similar way that it has been described for other organisms [8]. Additionally, it is logical to assume that some of these EhPRMTs are responsible of the epigenetic mark showed in Figures 1 and 2.

### 3D structures of EhPRMT1 proteins present homology with PRMT1

**Figure 4:**
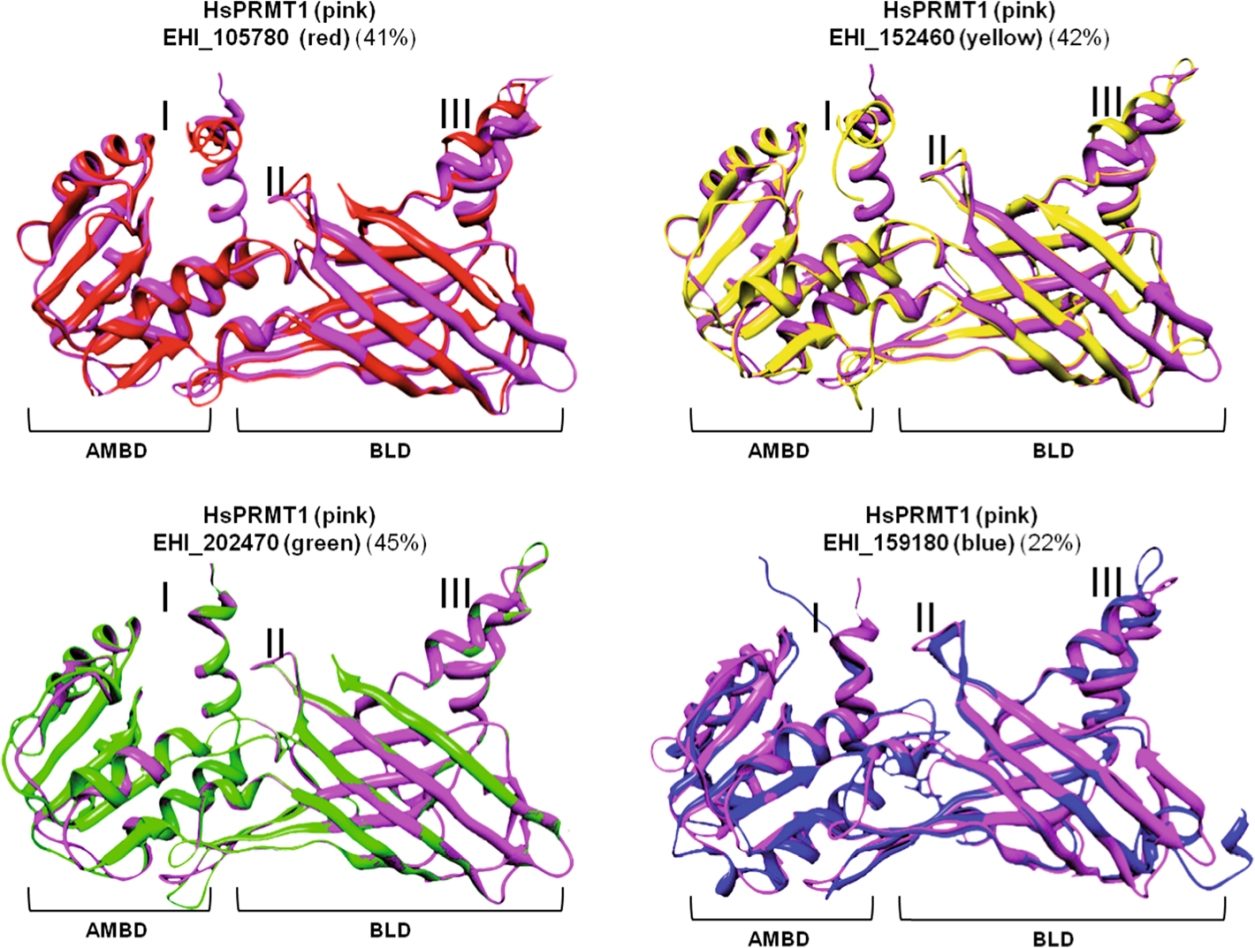
**3D Structure of EhPRMTs.** The 3D Structure of EhPRMTs was predicted using the Phyre2 program and the HsPRMT1 crystal as template. The percentages of structural homology to HsPRMT1 are indicated in parenthesis. (AMBD) AdoMet-binding domain. (BLD) Barrel-like structure. (I) Two amino-terminus helices. (II) Active site. (III) Dimerization arm.

To support the hypothesis that those proteins correspond to *E. histolytica* PRMT1 we predicted their 3D structures using the Phyre 2 program (http://www.sbg.bio.ic.ac.uk/phyre2/html) and the human PRMT1 (HsPRMT1) crystal as a template. Predicted 3D structures showed that the EhPRMT1-like proteins have a structural homology from 22 to 45% with HsPRMT1 (Figure 4). We identified in the four EhPRMTs the AdoMet-binding domain (AMBD) and the barrel-like structure domain (BLD) (Figure 4). We also localized in the 3D structures the two amino-terminus helices in AMBD (Figure 4, domain I), the active site formed by the double E loop and the THW loop in BLD (Figure 4, domain II), and the dimerization domain in BLD (Figure 4, domain III). Results showed that these EhPRMTs have the PRMT typical domains.

### EhPRMTs are phylogenetically related with human PRMTs

**Figure 5:**
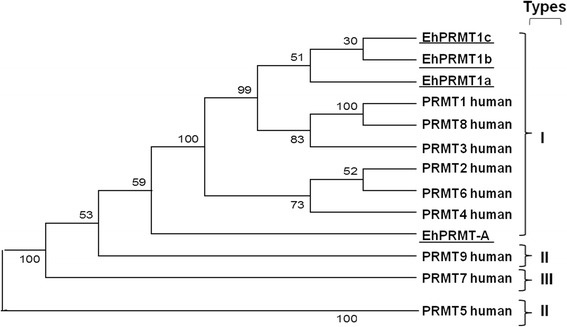
**Phylogenetic comparison of EhPRMT proteins.** The predicted amino acid sequences of the putative EhPRMTs were aligned with sequences of human PRMTs by ClustalW and data were submitted to phylogenetic analysis by UPGMA using MEGA version 5.05. Protein sequences used for this analysis were: Human PRMT proteins: PRMT1 (Q99873), PRMT2 (P55345), PRMT3 (O60678), PRMT4 (Q86X55), PRMT5 (O14744), PRMT6 (Q96LA8), PRMT7 (Q9NVM4), PRMT8 (Q9NR22) PRMT9 (Q86XK2); *E. histolytica* PRMT proteins: EhPRMT1a (EHI_105780); EhPRMT1b (EHI_152460); EhPRMT1c (EHI_202470); and EhPRMT-A (EHI_159180). Accession numbers are from GenBank®. Numbers at the branch nodes indicate the confidence percentages of the tree topology from bootstrap analysis of 1000 replicates. Proteins are grouped in I, II, and III enzymatic types.

We carried out a comparison of the EhPRMT1-like proteins with human PRMTs to analyze their phylogenetic relationship, and consequently, to obtain further evidence that those EhPRMTs could be the orthologs of HsPRMT1. The EHI_159180 sequence (EhPRMT-A) displayed limited association with other HsPRMTs; it showed a near relationship with type I enzymes, but also with HsPRMT9, which performs type II activity (Figure 5). However, it has no tetratricopeptide repeats found in HsPRMT9 [26]. Due to the low homology of this protein with other known PRMTs, we named it as an atypical PRMT protein (EhPRMT-A). On the other hand, EHI_105780 (EhPRMT1a), EHI_152460 (EhPRMT1b), and EHI_202470 (EhPRMT1c) sequences showed a nearness relationship to type I enzymes, specifically to HsPRMT1, HsPRMT3 and HsPRMT8 (Figure 5). These *E. histolytica* PRMTs do not contain the C2H2 type zinc-finger domain present in HsPRMT3 [26,27], nor the characteristic signal of myristoylation present in the N-terminus of HsPRMT8 [26]. This suggests that these three *E. histolytica* proteins are orthologs to HsPRMT1, which methylates H4R3 and other non-histone proteins [26,28]. Consequently, we named these proteins as EhPRMT1a, EhPRMT1b, and EhPRMT1c (Figure 5), which showed 37/42, 37/46 and 41/48% identity/similarity to the sequence of human PRMT1, respectively [18]. This analysis led us to suggest that one or more of these EhPRMT1 proteins are responsible to achieve the epigenetic mark that we detected in Figures 1 and 2.

### EhPRMT1 proteins are recognized by an antibody against HsPRMT1

**Figure 6:**
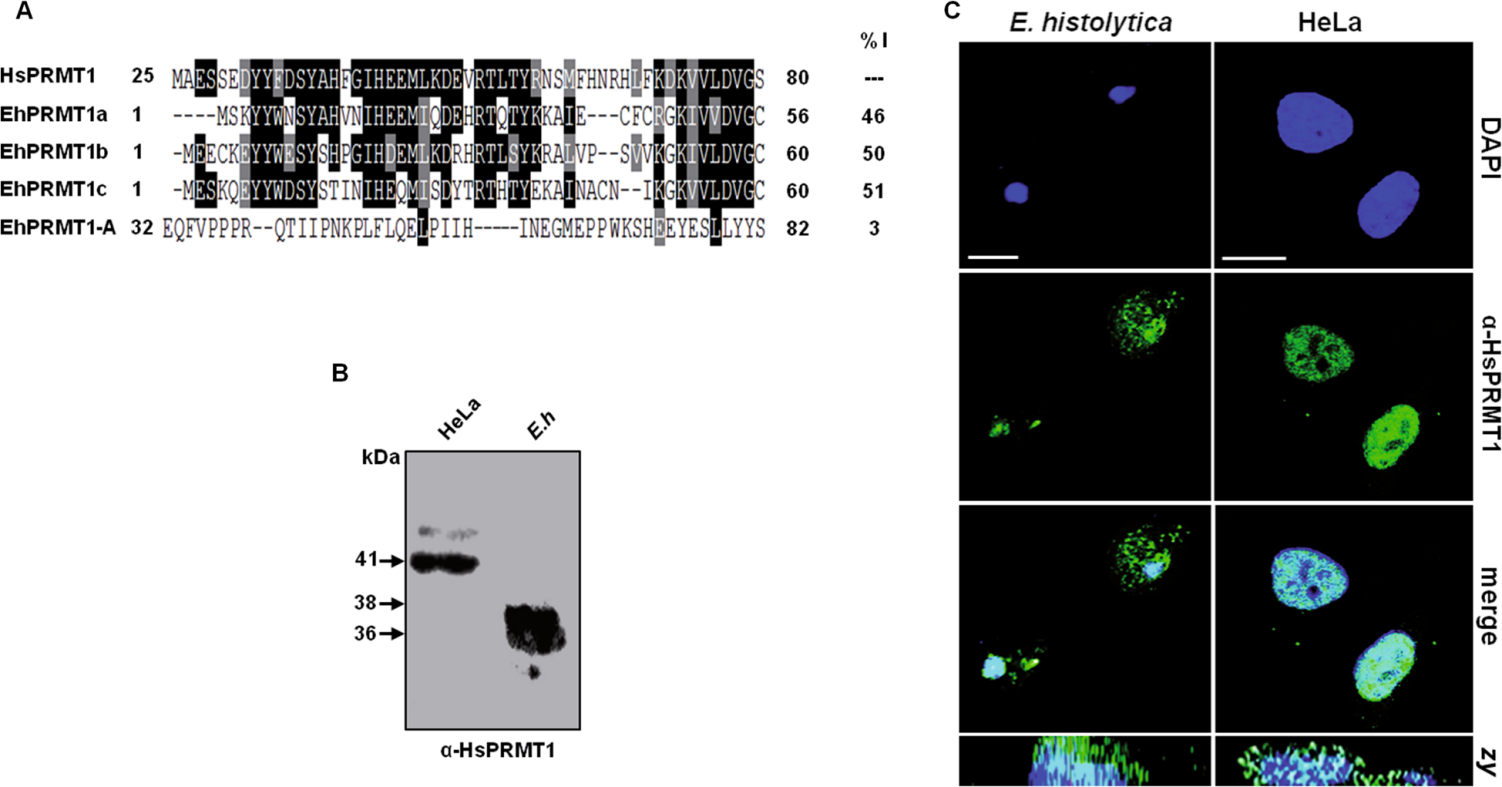
**Detection and localization of EhPRMT1 proteins. (A)** Alignment of the N-termini of EhPRMT1a, EhPRMT1b, EhPRMT1c, and EhPRMT-A with the corresponding fragment of HsPRMT1. Identical and similar residues to human PRMT1 are indicated in black and grey, respectively. %I, percentage of identity with the N-terminus of HsPRMT1 **(B)** Western blot assay on *E. histolytica* extracts using the α-PRMT1 commercial antibody. HeLa cells: positive control. **(C)** Confocal immunofluorescence assays on *E. histolytica* and HeLa cells using the α-PRMT1 antibody and subsequently a FITC-labeled secondary antibody (green). Nuclei were stained with DAPI (blue). *Zy-plane*: lateral images of the cells. Bar scale = 10 μm.

To support the hypothesis that EhPRMT1a, EhPRMT1b and EhPRMT1c are orthologs of HsPRMT1*,* we used a commercial antibody against human PRMT1 (α-PRMT1) that was generated against the first 52 amino acids of the N-terminus of HsPRMT1. We first aligned the HsPRMT1 N-terminus with the N-termini of the three EhPRMT1 to find out their homology with the antigen used to produce the commercial antibody. In this alignment we also included the N-terminus sequence of EhPRMT-A. This analysis showed that in this region EhPRMT1a, EhPRMT1b, and EhPRMT1c maintained 24 (46%), 26 (50%) and 27 (51%) identical amino acids, respectively, to HsPRMT1, while EhPRMT-A displayed poor homology in this region (3%) (Figure 6A). Thus, the α-PRMT1 antibody may recognize the three EhPRMT1 proteins. To confirm this, we analyzed by Western blot assays the ability of α-PRMT1 to detect the EhPRMT1 proteins. Our experiments showed that this antibody recognized two to three bands from 37 to 38 kDa in *E. histolytica* extracts (Figure 6B). The predicted molecular weight for EhPRMT1a, EhPRMT1b, and EhPRMT1c are 38.1, 37.5, and 36.6 kDa, respectively. Their close molecular weight make it hard to distinguish the recognition of each EhPRMT1 in these assays, but according with their homology at sequence and structure level it is possible to suggest that the antibody recognizes the three EhPRMT1 proteins. In HeLa cells extracts, used as a positive control, the antibody recognized a 41 kDa band (Figure 6B), the expected molecular weight for the human PRMT1 enzyme.

Laser confocal experiments using the α-PRMT1 antibody, allowed us to localize EhPRMT1 proteins in nuclei, as well as in cytoplasm of trophozoites (Figure 6C). Similar fluorescent patterns were observed in HeLa cells, used as a positive control (Figure 6C), suggesting that EhPRMT1 proteins could have similar roles as the human PRMT1, that is, to methylate H4 histone and possibly other cytoplasmic proteins [26].

### The recombinant EhPRMT1a protein is recognized by the α-PRMT1 antibody

**Figure 7:**
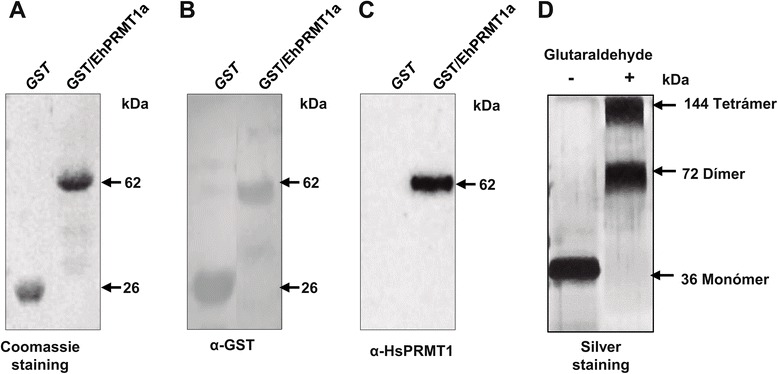
**Detection of the recombinant GST/EhPRMT1a by α-PRMT1 and oligomerization of EhPRMT1a. (A-C)** Purified GST and GST/EhPRMT1a recombinant proteins were separated in 12% SDS-PAGE and transferred to nitrocellulose filters. **(A)** SDS-PAGE stained with Coomassie blue. **(B, C)** Western blot assays using **(B)** α-GST or **(C)** α-HsPRMT1 antibodies. **(D)** Recombinant EhPRMT1a without the GST tag was incubated in the absence (−) or in the presence (+) of 0.2% glutaraldehyde for 20 min at 25°C prior to SDS-PAGE and silver staining. Bands corresponding to monomers, dimers and tetramers are indicated.

To corroborate that α-PRMT1 recognizes the EhPRMT1 proteins, we arbitrarily selected the EhPRMT1a protein to be cloned and expressed, fused to GST (GST/EhPRMT1a), in *E. coli*. Then, purification of GST/EhPRMT1a was carried out using glutathione beads. In Coomassie blue stained gels we observed a single 62 kDa band in the purified fraction (Figure 7A). This molecular weight corresponds to that expected for the recombinant protein (26 kDa from GST and 36 kDa from EhPRMT1a). Besides, an antibody against the GST tag (α-GST) detected the band corresponding to the recombinant GST/EhPRMT1a (62 kDa) and the 26 kDa of GST alone, used as a control (Figure 7B). Correspondingly, the α-PRMT1 antibody detected the recombinant GST/EhPRMT1a protein (Figure 7C), confirming that α-PRMT1 indeed recognizes the EhPRMT1a as an ortholog of human PRMT1.

Several PRMT1 family members are known to form homodimers or higher-order homo-oligomers, and it has been described that oligomerization is critical for the catalytic activity of some human PRMTs [29,30]. PRMT1 of *Trypanosoma brucei* and *Plasmodium falciparum* are also able to assemble into multimeric forms [31,32]. Here, we showed that the dimerization arm of human PRMT1 overlaps with a similar structure of EhPRMT1a (Figure 4, domain III), suggesting that the *E. histolytica* enzyme could be able to form homodimers. To analyze whether EhPRMT1a forms homo-oligomers, the purified recombinant protein, without the GST tag, was incubated in the presence of the cross-linking agent glutaraldehyde. Results, analyzed by SDS-PAGE, showed that the untreated EhPRMT1a migrated in a band of 36 kDa, the size predicted for a monomeric form, whereas the cross-linked protein displayed bands corresponding to the size of homodimers (72 kDa), and homotetramers (144 kDa) (Figure 7D). These results suggest that EhPRMT1a is able to assemble into multimeric forms.

### The recombinant EhPRMT1a has methyltransferase activity

**Figure 8:**
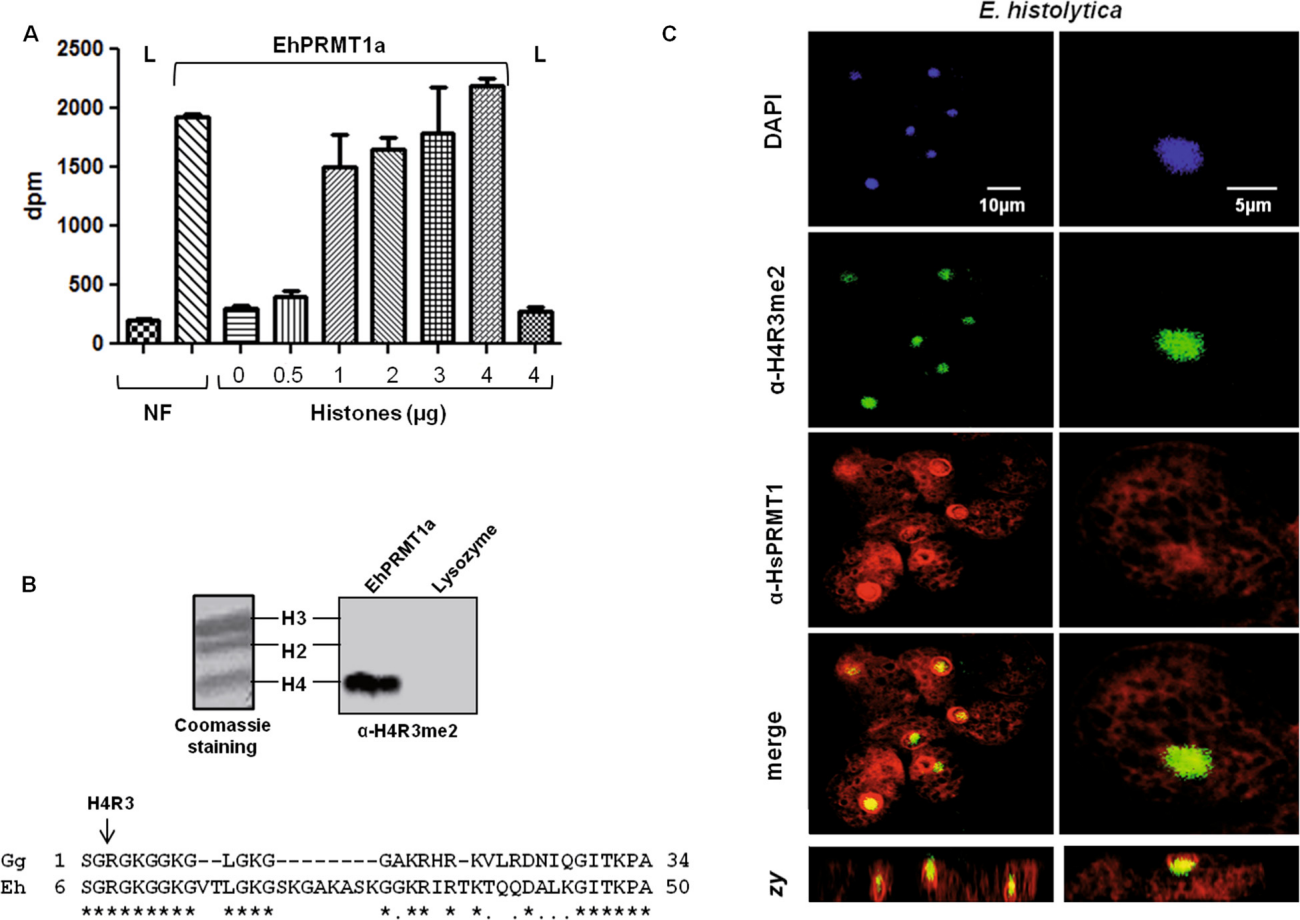
**Enzymatic activity ofEhPRMT1a and co-localization of EhPRMT1 and EhR8me2. (A)** Recombinant EhPRMT1a (1 μg) was incubated with10 μg of the nuclear basic fraction of *E. histolytica* trophozoites (NF) or with 0 to 4 μg of chicken histones in the presence of [^3^H]AdoMet and incorporation of radioactivity on the substrates was analyzed by scintillation counter. As a negative control, 1 μg of lysozyme (L) was incubated with 10 μg of the nuclear basic fraction or with 4 μg of chicken histones. Results are showed as the mean value ± standard deviation of three assays performed by duplicate. **(B)** 1 μg of recombinant EhPRMT1a or lysozyme (negative control) were incubated with 4 μg of chicken histones in the presence of non-radioactive AdoMet. Then, substrate specificity of EhPRMT1a was analyzed by Western blot using α-H4R3me2. Left: 15% SDS-PAGE of chicken histones stained with Coomassie blue. Right: Western blot showing the detection of H4R3me2. Under is shown the alignment of the N-termini of chicken (Gg) and *E. histolytica* (Eh) H4 histones. (*) Identical residues. (**.**) Similar residues. Arrow indicates the arginine residue modified by PRMT1. **(C)** α-H4R3me2 and α-PRMT1 antibodies were labeled with Pacific blue 410 (green) and Alexa fluor 647 (red) respectively. Then, *E. histolytica* trophozoites were processed for immunofluorescence assays using these antibodies. Nuclei were stained with DAPI (blue). Right panels show a magnification of a single trophozoite. *Zy* planes, lateral images of the cells.

To investigate whether the recombinant EhPRMT1a has enzymatic activity, we incubated 1 μg of the purified recombinant enzyme with a histone-enriched nuclear fraction of trophozoites, in the presence of [^3^H] AdoMet. Then, we measured the level of radioactivity into that fraction. These assays showed that recombinant protein transferred tritiated methyl groups to *E. histolytica* nuclear proteins, whereas no significant radioactivity was incorporated when the histone-enriched fraction was incubated in the presence of lysozyme (Figure 8A). These results suggest that *Entamoeba* histones, and specifically EhH4, are possibly substrates for EhPRMT1a. To confirm that this enzyme methylates histones, we performed the activity assays on commercial chicken histones. Results demonstrated that methylation of histones occurs since 1 μg of substrate and the maximum of activity was achieved with 4 μg of histones (Figure 8A). No significant radioactivity was detected when we incubated 4 μg of histones in the absence of recombinant enzyme or when they were incubated with 1 μg of lysozyme, used as a non-related protein (Figure 8A). These results corroborate that EhPRMT1a has histone methyltransferase activity.

Subsequently, to determine whether EhPRMT1a methylates the arginine 3 of H4 histone, we carried out the enzymatic assays using the mixture of chicken histones and non-radioactive AdoMet. Then, reactions were analyzed by Western blot using the α-H4R3me2 antibody. In these assays, the band corresponding to the H4 histone was recognized by the antibody, whereas no bands were detected when we incubated the histones with lysozyme (Figure 8B). These results indicate that EhPRMT1a catalyzes the dimethylation of H4R3 in commercial histones.

Finally, to analyze if there is a relationship between EhH4 and EhPRMT1 we carried out immunofluorescence assays using the α-PRMT1 and α-H4R3me2 antibodies. These experiments showed that EhPRMT1 and EhH4R8me2 co-localize in the trophozoite nuclei (Figure 8A), supporting the assumption that EhH4 could be a substrate for EhPRMT1.

## Discussion

The different virulence degree displayed by *E. histolytica* trophozoites under diverse environmental conditions could be due to changes in expression of certain genes [33-36]. In addition, the life cycle of this parasite involving the reversible conversion of infective forms (cysts) to invasive cells (trophozoites) is expected to be also due to differential expression of *E. histolytica* genes. However, the molecular mechanisms that are involved in the expression regulation of this parasite are poorly comprehended.

Similar to other eukaryotes, epigenetics may play an important role in the gene expression of this parasite. *E. histolytica* chromatin is packaged in nucleosome-like structures; however, linker regions between adjacent nucleosomes appear to be irregular in length [11]. In addition, the amino-termini of *E. histolytica* histones diverge from metazoan sequences, but these regions are highly basic and contain several arginine and lysine residues that are potential targets for acetylation and methylation, modifications that may modulate gene expression [13,37].

Indeed, it has been shown that demethylation of H3K4 is associated to transcriptional inactivation in this parasite [16]. Besides, some histone acetyltransferases and histone deacetylases have been identified in *E. histolytica* [15], and a protein containing a SET domain, which is typical of enzymes that add methyl marks on lysines of histone tails, was detected in the cyst stage of this protozoa [38]. These results suggest that the epigenetic machinery has an important role in growth and development of *E. histolytica*.

According with this statement, in this study we present evidence that the H4 histone of this microorganism (EhH4), in spite of its amino-terminus divergence with respect to the consensus H4, contains arginine and lysine residues that align with those that are methylated in H4 of other eukaryotes. The EhH4R8 residue, equivalent to the H4R3 of the consensus protein, was recognized by an antibody against the dimethylated H4R3 (α-H4R3me2). Arginine methylation is achieved by enzymes known as PRMTs. Thus, the recognition of EhH4 by the α-H4R3me2 antibody indicates the presence of active PRMTs in *E. histolytica*.

PRMT genes are present in eukaryotes from fungi to animals, and multiple genes have been found in plants and animals. *E. histolytica* contains five putative PRMT genes [18]. Although this parasite is an early branching eukaryote, one of the five EhPRMTs displays high amino acid conservation with PRMT5, and three EhPRMTs are homologous to PRMT1. In our phylogenetic and structural analyses, the other EhPRMT displayed limited association with other PRMTs and we named this enzyme as EhPRMT-A. This protein showed some degree of phylogenetic relationship to human PRMT9, which performs PRMT type II activity. Interestingly, mammalian PRMT9 also displays limited sequence homology with other PRMTs [2,3]. Therefore, it is possible that EhPRMT-A, in spite of the absence of the typical tetratricopeptide motif of PRMT9, possesses a type II PRMT activity. However, this hypothesis must be experimentally tested.

In mammals, the H4R3 residue is methylated by PRMT1 [28] and our phylogenetic analysis suggested that *E. histolytica* contains three PRMT1 proteins. In addition to the sequence similarity of EhPRMT1a, EhPRMT1b, and EhPRMT1c with human PRMT1, structural homology of these proteins with HsPRMT1 is evident in each of the four distinct PRMT domains: the N-terminus domain, the AdoMet binding domain, the dimerization arm, and the β-barrel domain, strongly suggesting that they could be the orthologs of PRMT1.

The N-termini of EhPRMT1 proteins showed significant homology to the same region of HsPRMT1, which is recognized for the α-PRMT1 antibody. In Western blot assays on *E. histolytica* extracts, this antibody recognized two to three bands from 37 to 38 kDa; however, due to their relatively close molecular weight we cannot distinguish each EhPRMT1. Nevertheless, our RT-PCR assays indicated that all *Ehprmt1* genes are expressed in trophozoites, suggesting that the three enzymes could be recognized by the antibody.

On the other hand, the activity assays performed with the recombinant EhPRMT1a showed that it catalyzes the transference of methyl groups to the chicken H4 histone, suggesting that at least this enzyme has similar substrates to HsPRMT1.

The presence of several PRMTs type 1 in *E. histolytica* could suggest that they possibly play a synergistic cooperation as one PRMT1 adds the first methyl group while another PRMT1 incorporates the second methyl group. However, the antibody used in this work specifically recognizes the dimethylated status of H4R3, therefore the antibody recognition of H4 on chicken histones incubated with recombinant EhPRM1a strongly suggests that this enzyme is able to catalyze the incorporation of both methyl groups. However, this hypothesis should be experimentally tested. Further assays using the recombinant proteins of EhPRMT1b and EhPRMT1c might help to elucidate if these enzymes have redundant activities or whether they have non-overlapping functions, as it occurs with PRMT3 and PRMT8 of vertebrates, which are very similar in sequence to PRMT1, but have different substrates [39,40].

Methylation of arginine residues in histones has profound effects on gene expression [41]. In this study we showed the presence of methylated H4R8 in *E. histolytica* trophozoites and we proved that the recombinant EhPRMT1a enzyme methylates the chicken H4. We also showed that EhPRMT1s and EhH4R8me2 co-localize in nuclei of trophozoites. Thus, possibly EhPRMT1a catalyzes the dimethylation of EhH4R8, even though further experiments using recombinant *E. histolytica* histones as substrate will confirm this assumption. Although the methylation of H4R3 is known to be involved in transcription activation in mammals [42], the significance of methylation of EhH4R8 in the epigenetics of *E. histolytica* awaits further elucidation.

In addition to histones, several proteins had been identified as substrates of PRMT1, including RNA-binding proteins, ribosomal proteins and transcription factors [2]. Methylation of these proteins could also impact upon gene expression. For example, the *Toxoplasma gondii* PRMT1 methylates H4R3, but it also catalyzes the methylation of the argonaute ortholog to regulate the recruitment of a Tudor staphylococcal nuclease (TSN), a more potent second slicer to perform the parasitic RNA silencing [43]. Interestingly, an *E. histolytica* TSN was identified as the transcription factor that binds to the *cis*-regulatory sequence URE1 (EhURE1-BP) [44], suggesting that probably the methylation of non-histone substrates could affect the transcriptional activity of EhURE1-BP. Future investigations to search the substrates of EhPRMT1 proteins may reveal novel pathways in which these enzymes play regulatory roles in this parasite.

## Conclusion

In conclusion, in this study we demonstrated the presence of methylated H4R8 in *E. histolytica* trophozoites (equivalent to H4R3 of other eukaryotes), which perhaps participates in the epigenetic regulation in this parasite. In addition, we identified three EhPRMT1 proteins, and proved that they, or at least one of them, co-localize with H4R8me2 in the nuclei of trophozoites. Furthermore, the EhPRMT1a recombinant protein is able to transfer methyl groups to arginine 3 of chicken H4 and to a histone-enriched fraction of *E. histolytica*, suggesting that this enzyme possibly methylates the arginine 8 of EhH4 and this modification must be involved in the epigenetics of *E. histolytica*.

## Abbreviations

AdoMet: S-adenosyl methionine
AMBD: AdoMet-binding domain
BLD: Barrel-like domain
GST: Glutathion-S-transferase
H4R3: Arginine 3 of histone H4
H4R3me2: H4R3 dimethylated
H4R8: Arginine 8 of histone H4 of *E. histolytica*
PRMT: Protein arginine methyltransferase
EhPRMT: PRMT of *E. histolytica*
HsPRMT: Human PRMT
